# The Use of Membranes (ST-100, oXiris, and M60) for Continuous Renal Replacement Therapy in a Child with Sepsis

**DOI:** 10.1155/2023/2000781

**Published:** 2023-06-06

**Authors:** Jiayun Ying, Xiaodi Cai, Guoping Lu, Weiming Chen

**Affiliations:** Pediatric ICU, Children's Hospital of Fudan University, Shanghai, China

## Abstract

Sepsis is a critical condition affecting patients worldwide. Systemic inflammatory response syndrome in sepsis contributes to organ dysfunction and mortality. The oXiris is a recently developed continuous renal replacement therapy (CRRT) hemofilter indicated for the adsorption of cytokines from the bloodstream. In our study, in a septic child, CRRT with three filters, including the oXiris hemofilter, resulted in a downregulation of inflammatory biomarkers and a reduction of vasopressors. Herein, we described the first report of such usage in septic children.

## 1. Introduction

Sepsis is a life-threatening organ dysfunction due to a dysregulated host response to infection and remains a common cause of death in critically ill children. A study showed that sepsis-related deaths were nearly 2.9 million among children younger than five years, 454 000 among children and adolescents in 2017 [1]. Although the morbidity and mortality of sepsis have been significantly reduced, severe sepsis-related deaths accounted for 9%-20% [2]. In septic shock, a cytokine storm performed as inflammatory mediators are produced and released, resulting in organ dysfunction and mortality. Removing cytokines from the blood can help mitigate inflammatory damage and prevent sepsis-related mortality.

Continuous renal replacement therapy (CRRT) is an extracorporeal life support to prevent or reduce fluid overload in septic children. For septic children with acute kidney injury (AKI), especially those with hemodynamic instability, CRRT is also recommended [3]. In recent clinical studies, blood purification has been described as removing cytokines through several new techniques, including therapeutic plasma exchange and hemoperfusion [4]. However, the efficacy of these methods in treating sepsis remains unclear.

Compared with the AN69ST hemofilter, the oXiris hemofilter enhanced the adsorptive properties to adsorb more cytokines and LPS. The oXiris membrane comprises three layers: heparin grafting, multiple polyethyleneimines (PEI) layers, and an AN69 copolymer hydrogel structure [5]. A growing body of literature recognizes the utility of oXiris hemofilter in patients with sepsis [6–8]. A study showed no difference in prognosis in patients treated with AN69-oXiris and those treated with the AN69-ST filter, but the former improved hemodynamic status and lower cytokine levels [9]. However, there is still little evidence to support using oXiris for sepsis, especially in children.

In this report, we present one case from the Children's Hospital of Fudan University to help identify the efficacy of the oXiris hemofilter in children with sepsis. The child was administered this treatment due to an abdominal cavity infection.

## 2. Case Description

**Figure 1:**
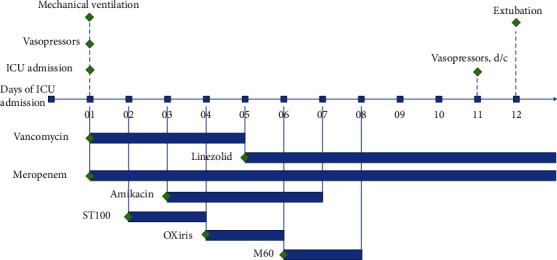
ICU treatment summary. d/c: discontinued; ICU: intensive care unit.

**Figure 2:**
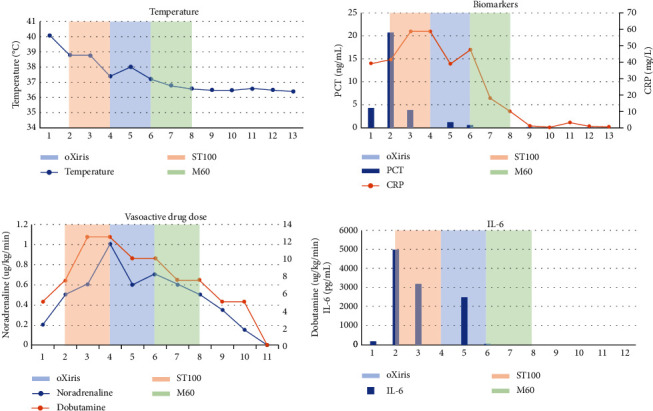
Changes in clinical and biochemical data before and after treatment with the oXiris® membrane. Body temperature, vasopressors doses, PCT, CRP, and IL-6 levels related to the use of the oXiris® membrane. CRP: C reaction protein; PCT: procalcitonin.

This report shows a 6-year-old boy (Table 1) weighing 20 kg with abdominal bacteremia-mediated septic shock who was admitted to the intensive care unit (ICU). He had a 3-day history of fever after exploratory laparotomy with abdominal incision exudate and erythema polymorphe. With the deterioration of symptoms, including fever (39°C), dyspnea, severe hypoxia, hypotension, and oliguria, he was diagnosed with septic shock, postoperative abdominal incision infection, and erythema multiforme. Supportive treatment (fluid resuscitation, antibiotic therapy, mechanical ventilation, norepinephrine, and dobutamine) was initiated and adapted from the surviving sepsis campaign-2016 guideline (shown in Figure 1). Due to the systemic inflammatory reaction, the patient had stage 2 acute kidney injury with urine output below 0.5 mL/kg/h for 15 h, continuous venovenous hemodiafiltration (CVVHDF) with ST100 membrane (Baxter, Deerfield, IL, USA) was initiated 14 hours each day for two days (Table 2). However, the patient's vasoactive inotrope score (VIS) raised to 112.5. Although broad-spectrum antibiotics vancomycin and meropenem were used and high doses of vasopressors were given, the patient still had cytokine storm with persistent fever. With the approval of the patient's family, we implemented an oXiris hemofilter in place of the ST100 filter to enhance the clearance of cytokines and endotoxins. The extracorporeal volume of the oXiris hemofilter and the circuit was 195 ml. So, at the onset of CRRT, the prefilled normal saline was emptied and refilled with colloidal fluid to prevent hypotension and blood dilution. We used 50 ml 5% albumin and 100 ml plasma for priming. Blood flow was slow at 45 ml/min at the beginning 10 min and then gradually increased. Heparin was used as an anticoagulation agent, and the patient's fluid balance was titrated at 1 h. This blood purification protocol was continued for 12 h each day for two days (Table 2). The inflammatory biomarkers and doses of vasopressors rapidly declined (shown in Figure 2 and Table 2). Because of partial AKI recovery, the patient received CVVHDF with an M60 hemofilter to treat fluid overload for 13 h one day and 14 h on another day (Table 2). Considering CRRT, the dose of vancomycin was adjusted to 10 mg/kg/times each 12 h, and the dose of meropenem was adjusted to 17.5 mg/kg/times each 12 h. After treatment, the patient was weaned off mechanical ventilation and transferred to the general ward from the ICU several days later. Finally, the patient stayed in ICU for 20 days with a total hospitalization duration of 31 days.

## 3. Discussion

In this study, we found that the oXiris hemofilter in the setting of CRRT could be clinically feasible in children with septic shock.

Blood purification therapies can remove cytokines and have been suggested to improve immune homeostasis in sepsis [10]. It attracted much attention for using extracorporeal adsorption to remove inflammatory mediators from the bloodstream [11, 12]. In 2018, a study showed that the hemodynamic and respiratory parameters were improved in ten children with sepsis after they received the lipopolysaccharide-adsorption course by hemoperfusion with PMX [13]. Also, a case series showed that it might be an efficacious and relatively safe adjunctive treatment for hemoperfusion with HA330 cartridges to clear the cytokine storm in children with sepsis [14]. So, extracorporeal adsorption is a prospective adjunctive therapy for children with sepsis.

The oXiris membrane has an enhanced adsorption property of cytokines and LPS [10]. This filter may address several therapeutic targets in sepsis through its multiple functions, including adsorption of endotoxin and cytokine and renal replacement treatment [11]. In a recent study, CRRT with the oXiris membrane was used in 60 patients with sepsis. It showed that CRRT with the oXiris membrane set improved patient outcomes with a reduction of vasopressors and a downregulation in blood inflammatory mediators [12]. Another study also found that CRRT with the oXiris membrane may decline the lactate level and infection indicators [13]. However, a study that enrolled 136 patients showed no difference in 90-day mortality in the oXiris group and the ST150 group, although the oXiris group had lower 7-day and 14-day mortality [14]. In contrast, a retrospective cohort study found that the oXiris-CVVH had lower 28-day mortality than the AN69 filter-CVVH group after the inverse probability of the treatment-weighting method [15]. No large RCT study has confirmed the improvement of oXiris in the prognosis of sepsis. There is little literature on the use of oXiris membrane in the pediatric population. A case report shows the use of oXiris membrane in managing multisystem inflammatory syndrome in a seven-year-old child after COVID-19. The cytokines were cleared, and the demand for vasopressors was reduced in patients with other inflammatory conditions [16]. In our report, cytokines and the need for vasopressors were reduced after treatment. Procalcitonin was decreased from 20.62 ng/ml to 0.11 ng/ml. C reaction protein was decreased from 58 mg/L to 1.27 mg/L. Moreover, the dose of noradrenaline was reduced from 1 ug/kg/min to 0.6 ug/kg/min. The dose of dobutamine was decreased from 12.5 ug/kg/min to 7.5 ug/kg/min. All in all, after using three filters (ST-100, oXiris, and M60), inflammation control could be achieved clinically. This is the 1st case of using such a combination of filters in a pediatric patient.

Although the oXiris has previously performed well in patients with sepsis, there are notable limitations in implementing its use to manage sepsis. The oXiris does not replace infection source control protocol which must be performed for patient recovery. When implementing oXiris, the plasma concentration of antibiotics may be lower than expected, which confounds efforts to designate the proper antibiotic dose. The system also faces unique limitations in pediatric patients. Children face more significant difficulties during sepsis treatment than adults due to their small blood volume. A case report showed that multisystem inflammatory syndrome occurred in a 14-year-old male after he acquired the SARS-CoV-2 infection. He received CRRT with a hemoadsoprtion column Cytosorb and a hemofilter oXiris. Then, the levels of several cytokines were reduced, and his condition improved [17]. However, in low-weight children, CRRT can cause hypotension due to the large capacity of extracorporeal circulation, which is a common complication of CRRT [18]. This can aggravate circulatory instability in patients with sepsis. Therefore, the patient should be primed with blood products or colloidal fluid at the onset of CRRT, and blood flow should be monitored carefully to ensure stable circulation. In this case, we used colloidal fluid for priming and reduced the blood flow initially. There was no technical problem with the use of oXiris in a 20-kg child.

## 4. Conclusion

For severely ill children diagnosed with septic shock, a potential management strategy is proposed from our experience. As far as we know, we described the first report of such usage in septic children. However, this is a single-case experience that requires further confirmation through randomized controlled studies in the future before making recommendations.

## Figures and Tables

**Table 1:** Patient demographics and clinical data.

Demographics	6-year-old boy

Weight (kg)	20

Height (cm)	113

Clinical diagnoses	Septic shock, postoperative abdominal incision infection, erythema multiforme

Initial vital signs	RR 35 b/min
	HR 139 b/min
	BP 63/37 mmHg
	MAP 45 mmHg
	SPO2 95%

Initial laboratory workup	PaO2 90 mmHg N: 83-108
	PaCO2 41.4 mmHg N: 35-45
	P/F 180 N: >300
	Lac 2.2 mmol/L N: 0.5-1.6

Initial therapeutic parameter	PC
	FiO2 50%
	PEEP 6 cmH2O
	PC above PEEP 18 cmH2O
	Ti 0.8 s
	RR 30 times/min

Vasoactive inotrope score	25

Broad-spectrum antimicrobials	Vancomycin, meropenem

Infection site	Abdomen

Pathogen	Not identified

PELOD-2	7 (respiration system 3 scores, mean artificial pressure 4 scores, platelet counts 1 score)

MODS	Cardiovascular dysfunction, respiratory, renal

Survival	Yes

Ventilation time (d)	10

Length of ICU stay (d)	18

Complications	No

PELOD-2: pediatric logistic organ dysfunction-2; MODS: multiple organ dysfunction; CRRT: continuous renal replacement therapy.

**Table 2:** The dynamics of clinical and laboratory parameters during CRRT.

Days after ICU admission	D1	D2	D3	D4	D5	D6	D7
Respiratory rate (b/min)	30	28	25	25	27	26	18
PEEP (cmH_2_O)	6	6	6	6	5	6	5
FiO_2_	50	60	60	50	40	35	35
PC above PEEP (cmH_2_O)	18	18	17	15	12	12	10
PaCO_2_ (cmH_2_O)	41.4	36	39.7	37.7	39.1	37.1	35.9
PaO_2_ (cmH_2_O)	90	105	114	120	128	115	111
P/F	180	175	190	240	320	328	317
Heart rate (b/min)	139	138	135	148	108	121	107
Mean arterial pressure (mmHg)	45	58	66	58	84	72	84
Noradrenaline (ug/kg/min)	0.2	0.5	0.6	1	0.6	0.7	0.6
Dobutamine (ug/kg/min)	5	7.5	12.5	12.5	10	10	7.5
Creatinine (*μ*mol/L)	20	16	45	42	27	34	34
Urea nitrogen (mmol/L)	2.78	1.98	4.5	3.4	2.9	7.12	7.69
Urea (ml/24 h)	250	610	780	890	1030	1060	1230
CRRT parameters							
Mode	/	CVVHDF	CVVHDF	CVVHDF	CVVHDF	CVVHDF	CVVHDF
Membrane	/	ST100	ST100	oXiris	oXiris	M60	M60
Duration (h)	/	14	14	12	12	13	14
Blood flow rate (ml/kg/h)	/	3	3	3.5	3.5	3.5	3.5
Replacement fluid rate (ml/kg/h)	/	30	30	40	40	40	40
Net ultrafiltration flowrate (ml/kg/h)	/	4	3	3	2	3	3

CRRT: continuous renal replacement therapy; CVVHDF: continuous venovenous hemodiafiltration; P/F ratio: PaO_2_/FiO2 ratio.

## Data Availability

The data used to support the findings of this study are included within the article.
